# Genetic associations of temperament and facial hair whorls with productive, reproductive, and carcass traits in Canchim beef cattle

**DOI:** 10.5713/ab.260151

**Published:** 2026-04-28

**Authors:** Ayrton Fernandes de Oliveira Bessa, Giovanna Maria dos Santos Câmara, Carolina Zucatelle, Felipe Kenji Futema, Roney Teixeira, Gabriela Cenedeze, Ana Luisa Paçó Bugner, Vanessa Aparecida Feijó de Souza, Walsiara Estanislau Maffei, Andrea Roberto Bueno Ribeiro, Cintia Righetti Marcondes, Marcos Eli Buzanskas

**Affiliations:** 1 Universidade Federal da Paraíba (UFPB), Areia, Brazil; 2 School of Veterinary Medicine and Animal Science (FMVZ), São Paulo State University (UNESP), Botucatu, Brazil; 3 School of Agricultural and Veterinarian Sciences (FCAV), São Paulo State University (UNESP), Jaboticabal, Brazil; 4 Centro Universitário das Faculdades Metropolitanas Unidas, São Paulo, Brazil; 5 Wairam Ltda., Uberaba, Brazil; 6 Universidade Santo Amaro, São Paulo, Brazil; 7 Embrapa Southeast Livestock, São Carlos, Brazil

**Keywords:** Animal Breeding, Behavior, Composite Breed, Pleiotropy

## Abstract

**Objective:**

This study aimed to estimate genetic parameters and evaluate genetic correlations between temperament and facial hair whorl traits with productive, reproductive, and carcass traits in Canchim beef cattle.

**Methods:**

Phenotypic records of animals born between 2013 to 2022 were analyzed. Temperament was assessed using behavioral scores, including movement, tension, breathing, and vocalization. Reactivity was measured using accelerometers and flight time, while facial hair whorls were characterized by their presence, number, position, and height. Traits related to productivity, reproduction, and carcass characteristics included body weights, scrotal circumference, and ribeye area at different ages. Genetic parameters were estimated using single- and two-trait animal models.

**Results:**

Heritability estimates ranged from low to high (0.09 to 0.67), indicating sufficient additive genetic variability for all trait groups. Positive genetic correlations were observed between body weight traits and temperament measures, particularly vocalization and flight time, in addition to correlations between ribeye area and reactivity flight time. Scrotal circumference at 12 and 18 months of age showed moderate to high genetic correlations with facial hair whorl traits, especially whorl number and position.

**Conclusion:**

The results indicate that selection for productive and reproductive traits may result in indirect improvements in temperament. Moreover, the pre-selection for the absence of facial hair whorls, along with the selection for body weight and scrotal circumference, may promote favorable genetic gains in temperament, supporting their inclusion into Canchim breeding programs.

## INTRODUCTION

Brazil plays a key role in the production of animal protein, being one of the largest beef producers in the world [1]. The country relies on important scientific and technological advances that have contributed to improving quality and sustainability. Beef cattle breeding programs are aimed at evaluating a set of traits according to the necessities of breeders and the consumer market and at identifying those traits that will compose the selection indexes according to their economic importance [2]. Such indexes mainly consider traits related to body weight and weight gain, reproductive precocity and longevity, and carcass quality [3].

In view of the concerns of society and producers in terms of animal welfare, studies on new traits have been conducted in order to evaluate the possibility of reducing injuries related to animal handling [4]. Within this context, the temperament of animals as a measure for assessing welfare comprises a broad set of traits that are related to and determine the behavioral responses of an individual. Therefore, temperament is evaluated based on the animal’s reaction to human interaction [5].

Studies indicate that animals that are less reactive to handling tend to be more productive [6]. In situations in which animals are more reactive, lower weight gain, poorer meat quality, susceptibility to disease, and low reproductive performance have been reported [7]. Furthermore, the presence of these animals in herds may increase production and labor costs, as well as the risk of accidents involving employees [8].

There are several approaches to assessing temperament in cattle, which consider the animal’s reactions to different external stimuli [9]. The most common temperament measures are obtained by the chute exit velocity, flight speed, flight time (FT) [10], and chute score [11]. Furthermore, studies reported the existence of an association between temperament and facial hair whorls. In cattle, temperament and facial hair whorls presented important genetic background, where facial hair whorl traits can contribute to the pre-selection of animals with desirable temperaments [9].

Grandin et al [12] showed that animals with hair whorls positioned above the eye line were more reactive to human presence. Taken together, the evidence indicates that both temperament and facial hair whorl traits possess a genetic component and may respond to selection [9,13]. Specifically in Canchim, a beef breed derived from crosses between Zebu (*Bos indicus*) and Taurine (*Bos taurus*) cattle [14], studies indicated that Zebu tend to exhibit higher excitability than *Bos taurus* cattle, and within *Bos taurus*, genetic variants of European Continental breeds (such as Charolais) have been reported to present higher excitability compared to British breeds [15]. Additionally, Charolais calves have been described as more nervous than calves of the Aubrac French beef cattle breed [16]. In a study involving crossbreeding between Nelore and Charolais cattle, Silveira et al [17] observed that animals with higher proportions of Nelore presented greater reactivity.

Therefore, to contribute to the Canchim breeding program, this study aimed to estimate genetic parameters for temperament, facial hair whorl, productive, reproductive, and carcass traits, and to evaluate the genetic associations of temperament and facial hair whorl traits with other traits.

## MATERIALS AND METHODS

The phenotypic data used belong to an experimental herd maintained by Embrapa Southeast Livestock, São Carlos/SP, Brazil. The Canchim cattle were developed in the 1940s, aiming to use crossbreeding between Charolais and Zebu cattle (mainly Nelore) to obtain animals adapted to the production system and climate of Brazil, resulting in a breed composition of 5/8 Charolais and 3/8 Zebu [14]. Further details on the history of the formation of the Canchim breed can be found in previous studies [14,18].

The studied animals were born between 2013 and 2022. The temperament traits were assessed based on scores [18], which were: movement (MOV: 1 = no MOV to 5 = animal jumping, raising its forelimbs at least 2.5 cm from the ground); tension (TEN: 1 = animal with regular muscle tonus, without sudden MOVs of the tail, head or neck, and a relaxed gaze to 4 = animal appears to be paralyzed, with muscle tremors); breathing (BRE: 1 = normal, rhythmic, poorly or non-audible BRE or 2 = blowing or puffing, with non-rhythmic BRE); and vocalization (VOC: 1 = absent or 2 = occurrence of mooing regardless of frequency or intensity).

Reactivity (REACT) was measured with the REATEST. This device is coupled to the weighing scale and uses accelerometers to rate the intensity and frequency of animal MOVs on a numerical scale ranging from 1 to 99,999 [19]. FT was obtained with the Duboi sensor, which measures the time, in hundredths of a second, that it takes an animal to traverse 1.83 meters after leaving the weighing scale. The animals were evaluated at 4 months of age, at weaning, at yearling, and at post-yearling. Temperament traits were measured on a weighing scale by trained evaluators.

Regarding the facial hair whorls, the lateral position was defined as absent (score 1) or present (score 2) (whorl on the left side [LW], whorl in the middle [MW], and whorl on the right side [RW]). The vertical position of facial hair whorls in relation to the eyes was defined as absent (score 1) or present (score 2) (low whorl [LoW], mid whorl [MidW], and high whorl [HiW]). The number of hair whorls (NW) was defined as absent (score 1) or present (scores 2 to 5 corresponding to one to four whorls, respectively). Whorl height (WH) was defined as absent (score 1) or present (scores 2 to 5 corresponding to low, medium, high, or very HiWs, respectively). Very HiW indicates the presence of the hair whorl in the upper part of the animal’s face, i.e., an area between the horns. The whorl trait (WHORL) was defined as absent (score 1) or present (score 2), regardless of the number or position of the whorls. The description and visualization of facial hair whorls were previously described by Bessa et al [9].

The following productive, reproductive, and carcass traits were studied: weaning weight (WW); body weight at 12 (W12) and 18 months of age (W18); scrotal circumference at weaning (SCW), scrotal circumference at 12 (SC12) and 18 months of age (SC18); and ribeye area at 12 (REA12) and 18 months of age (REA18). REA12 and REA18 were measured using a Piemedical Scanner 200 Vet equipped with an 18 cm linear transducer operating at 3.5 MHz, and an ALOKA 500V with a 17.2 cm linear probe at 3.5 MHz, positioned over the *longissimus dorsi* muscle between the 12th and 13th ribs.

Quality control of the phenotypic data was performed using the R program (https://www.r-project.org/foundation). A generalized linear model was used for the study of fixed effects. For REACT, the data were square-root transformed. The contemporary groups were formed considering combinations of the tested fixed effects (Supplement 1). Contemporary groups with fewer than three individuals and groups composed of offspring derived from only one sire and that did not show variability in the phenotypes, i.e., with only one of the scores, were excluded. The number of animals evaluated (Supplement 2) for temperament traits ranged from 1,231 (FT) to 2,061 (REACT). For whorl traits, 1,286 (WH) to 1,511 (NW) animals were considered. For productive, reproductive, and carcass traits (Supplement 3), 1,575 (W18) to 2,242 (WW), 545 (SC12) to 1,144 (SCW), and 771 (REA12) to 947 (REA18) animals were respectively considered.

The genetic parameters were estimated in single- and two-trait analyses under an animal model using the GIBBSF90+ program [20]. The matrix notation of the model for the temperament traits is represented by:


(1) y=Xβ+Za+WPe+e

in which *y* is the vector of observations; *β* is the vector of fixed effects; *a* is the vector of random additive genetic effects; *pe* is the vector of random permanent environmental effects; *X*, *Z* and *W* are incidence matrices that relate *β*, *a*, and *pe* to *y*, and *e* is the vector of random environmental effects. The variance of the permanent environment obtained with this model was used to estimate the repeatability.

The following model was used for WW and SCW:


(2) y=Xβ+Za+Mm+e

where *y* is the vector of observations; *β* is the vector of fixed effects; *a* is the vector of random additive genetic effects; *m* is the vector of random maternal additive genetic effects; *X*, *Z* and *M* are incidence matrices that relate *β*, *a* and *m* to *y*, and *e* is the vector of random environmental effects. The maternal additive genetic variance obtained with this model was used to estimate maternal heritability. For the other analyses (facial hair whorls, W12, W18, SC12, SC18, REA12, and REA18), random permanent environmental and maternal additive genetic effects were not considered. The relationship matrix included 10,308 animals.

For genetic parameter estimation, 1,100,000 iterations were considered, with a burn-in period of 100,000 iterations and a thinning interval of 500 iterations, totaling 2,000 samples for convergence analysis. The Geweke and Heidelberger and Welch convergence tests were performed using the boa package of the R software [21]. Convergence was established when the heritability, repeatability, and genetic correlation estimates met the criteria of one or both tests.

## RESULTS

The data structure of the facial hair whorls HiW and RW did not allow the genetic parameters to meet the convergence criteria and the results of these analyses will therefore not be reported. The other traits showed convergence in at least one of the tests used. The descriptive statistics of the traits studied are displayed in Supplements 2, 3. Estimates of direct heritability, maternal heritability, and repeatability estimates are presented in Table 1. The heritability estimates varied from 0.09 (BRE) to 0.67 (SC12), while the repeatability varied from 0.16 (BRE) to 0.55 (FT). Maternal heritability estimates were equal to 0.18 (WW) and 0.13 (SCW).

In Table 2, the genetic correlations that presented the highest posterior density range, which encompassed the estimate and presented the same sign, were between WW and VOC (–0.71), W12 and VOC (–0.71), WW and FT (–0.24), W12 and FT (–0.26), REA12 and REACT (–0.51), and REA12 and FT (–0.44). The highlighted phenotypic correlations (Table 3) among productive, reproductive, and carcass traits with temperament were between WW and VOC (–0.88), WW and FT (–0.15), W12 and VOC (–0.88), W12 and FT (–0.13), W18 and VOC (–0.91), and REA12 and FT (–0.21). SC12 and SC18 presented genetic correlations with MW, MidW, and NW (Table 4) ranging from −0.74 to −0.41. In Table 5, phenotypic correlations between productive, reproductive, and carcass traits with facial hair whorl traits varied from −0.44 to 0.14 but did not present estimates within the highest posterior density range. Genetic correlations between productive, reproductive, and carcass traits are presented in Supplement 4.

## DISCUSSION

Except for BRE, the heritability estimates for the temperament traits (Table 1) were classified as moderate to high, suggesting that these traits will respond satisfactorily to selection and less reactive offspring are expected. On the other hand, the repeatability of these traits indicated some consistency in the expression of the phenotypes measured at different ages, especially for FT. Thus, the temperament of Canchim cattle is influenced by genetic and permanent environmental components. The behavior of young animals tends to change gradually, possibly because of cumulative exposure to the stimuli presented; hence, for selection purposes, the collection of information at different ages is necessary [22].

Other studies reported heritability estimates for MOV, TEN, BRE, VOC, REACT, and flight speed of 0.17 [23], 0.07±0.04 [24], 0.04±0.01 [25], 0.71±0.03 [26], 0.39 [27], and 0.33±0.03 [28], respectively. These divergences in the results compared to the present study are related to differences in the breeds used and in the assessment methods of the animals.

The heritability estimates for the facial hair whorl traits ranged from low (0.18±0.04 for NW) to high (0.57±0.12 for LW and LoW). Overall, hair whorl traits in the Canchim breed showed sufficient genetic variability to respond to selection. Although these traits appear to be similar, differences were observed in genetic variability for height, position, and number of whorls. One of the advantages of evaluating facial hair whorls in cattle is that these traits are easy to obtain; they can be evaluated shortly after the animal is born and only require one observation, facilitating the implementation of these assessments on farms.

In Holstein cattle, heritability estimates of 0.54±0.08 and 0.11±0.06 were obtained for WH and whorl position in relation to the midline of the face, respectively [29]. Hair whorls are more frequently studied in horses and heritability estimates of 0.88±0.03 and 0.99±0.02 have been reported for position and number of facial hair whorls, respectively [13]. Cruz et al [13] found that animals with a larger number of hair whorls tend to be shyer during taming. For whorl position and NW, heritability estimates equal to 0.75±0.05 [30] and 0.66±0.097 [31] were observed in horses. The differences in the heritability estimates between studies conducted in horses and cattle can be attributed to variations in the statistical models applied (e.g., Cruz et al [13] and Górecka et al [30] did not include fixed effects), disparities in sample size (e.g., Shirley et al [29] and Yokomori et al [31] evaluated 470 cattle and 4,024 horses, respectively), as well as differences inherent to the species or breeds analyzed.

The high direct heritability estimates for body weights indicated that animals are likely to respond to direct selection, especially for W12. For SCW, SC12, and SC18, direct heritability estimates of 0.24±0.10, 0.67±0.15 and 0.53±0.12, respectively, were obtained, indicating that greater responses to selection can be expected for SC12. In Canchim animals, heritability estimates of 0.31±0.03, 0.29±0.02, and 0.28±0.01 were obtained for WW, W12, and W18, respectively [32].

In Nellore cattle, estimates of 0.31±0.03 and 0.38±0.02 were obtained for SC measured at 365 and 450 days of age, respectively [33]. The maternal heritabilities for WW and SCW suggest that the maternal genetic component plays an important role in the expression of these traits. The results observed agree with those reported by Vargas et al [34]. These authors highlighted the need for incorporating maternal effects in genetic parameter estimation models for pre-weaning traits to reduce bias. The heritability estimates for REA12 and REA18 were equal to 0.48±0.12 and 0.36±0.09, respectively, but were lower than those obtained by Tamagawa et al [35]. Thus, selection for these traits is expected to promote satisfactory genetic gains.

As can be seen in Tables 2, 4, a large part of the genetic correlation estimates was of low magnitude and had high standard deviations, indicating the absence of associations between most traits. One explanation for this observation could be the number of phenotypes; it is therefore recommended to continue these measurements to reduce potential bias in future studies of these traits in the Canchim breed.

Negative genetic correlations of WW and W12 with VOC and FT were observed, indicating that lower body weight performance is associated with higher VOC scores and a shorter time at which the animal leaves the scale and traverses 1.83 meters. This result suggests the existence of an additive genetic component that acts favorably on these traits, i.e., animals with higher WW and W12 are expected to have a desirable temperament in terms of VOC and FT, being calmer during handling in the pen. Animals with slower flight speed exhibited greater body weight gains (mean of 1.54 kg/day) than animals with faster flight speed (mean of 1.37 kg/day) [36]. In a study with Charolais and Nellore, animals with slower FT, i.e., that traverse a certain distance more slowly, had greater weight gain [17].

The genetic correlations of REA12 with REACT and FT were negative and of moderate magnitude. This result suggests that more reactive animals and animals with faster FT have a smaller ribeye area. Furthermore, the genetic correlations of WW and W12 with REA12 (Supplement 4) suggest the existence of a genetic component shared between these traits, indicating that they affect each other and that selection for one will indirectly and favorably affect the others.

According to Nkrumah et al [37], temperament influences carcass traits in beef cattle, highlighting the importance of including temperament in the definitions of selection objectives, as well as the culling of animals that are difficult to handle and the use of appropriate facilities to improve the handling experience of animals. Furthermore, Coutinho et al [38] observed that higher chute score and exit velocity were correlated with shear force and tenderness of the *longissimus lumborum* muscle, indicating that calmer animals produce better quality meat.

Other studies on cattle reported genetic correlations of scrotal circumference with flight speed and temperament scores of −0.28±0.02 [39] and −0.07±0.03 [40], respectively. According to Brandão and Cooke [5], animals with a more reactive temperament exhibit poorer reproductive performance than calmer animals; this fact can affect traits such as age at first calving of females, which also shows high genetic correlations with scrotal circumference of males.

The phenotypic correlations between productive, reproductive and carcass traits with temperament traits showed estimates that were mostly negative (Table 3). Favorable and high phenotypic correlations were observed between VOC with WW, W12, and W18 indicating that there is an association and that the variation in the expression of these traits in cattle is probably influenced by other environmental factors. A negative phenotypic correlation was also observed between FT with WW, W12, and REA12.

The phenotypic correlations for MOV and flight speed with body weight in Nellore animals were previously reported by Sant’Anna et al [24]. The authors observed that these correlations showed negative and low magnitude estimates of −0.05±0.01 and −0.08±0.01, respectively, which reflects a weak relationship between the traits. In crossbred animals, Burrow [41] observed phenotypic correlations between body weight and flight speed that ranged from −0.02 to −0.05, corroborating the results observed in our study. These same authors also observed phenotypic correlations for flight speed and scrotal circumference at different ages that ranged from 0.07 to 0.11.

In Table 4, only SC12 and SC18 showed important genetic correlations with MW, MidW, and NW. These results demonstrate that the position and number of facial hair whorls of the animal can influence male reproductive traits. Therefore, the genetic correlations of SC12 and SC18 with the highlighted whorl traits indicate that the genes responsible for the presence of whorls in these regions are also responsible for undesirable reproductive performance in males. In a study with Angus cattle, Meola et al [42] found that animals with a hair whorl pattern classified as round epicenter had a higher percentage of normal spermatozoa than animals with a non-round (or misshapen) epicenter. Investigating WH in beef cattle, Silveira et al [17] observed negative correlations with weight gain and FT. In contrast, Olmos and Turner [43] found no correlations of whorl position with weight gain or flight speed in beef cattle.

The phenotypic correlations between the productive, reproductive and carcass traits with the whorl traits are presented in Table 5, where they were observed as being of low magnitude, accompanied by high standard errors. This indicates that the phenotypic interactions between these estimates are not very relevant, suggesting a weak or inconsistent relationship between them. In general, the genetic correlations between productive, reproductive, and carcass traits were of moderate to high magnitude (Supplement 4), suggesting that a set of genes with additive effect act simultaneously and favorably on these traits. The genetic correlations of WW with SC12, SC18, and REA18; of SCW with REA12 and REA18; of SC12 with REA18; and of SC18 with REA18 were of low magnitude and had high standard deviations.

## CONCLUSION

This study showed that most of the traits exhibit additive genetic variability that can be explored by the Canchim breeding program, aiming at selecting animals with adequate performance and temperament. Genetic associations were observed between body weight traits with temperament and between scrotal circumference and carcass traits with facial hair whorls. Thus, pre-selecting animals for the absence of facial hair whorls, in conjunction with selection for W12 and SC12, may result in indirect and favorable genetic gain in offspring temperament.

## Figures and Tables

**Table 1:** Genetic parameter estimates and highest density intervals (in brackets) for the traits studied in Canchim cattle

Temperament			Facial hair whorl		Productive, reproductive, and carcass		

Traits	*h* ^2^ *_d_*	t	Traits	*h* ^2^ *_d_*	Traits	*h* ^2^ *_d_*	*h* ^2^ *_m_*
MOV	0.20±0.04 (0.11;0.28)	0.35±0.02 (0.31;0.39)	LW	0.57±0.12 (0.33;0.81)	WW	0.10±0.03 (0.03;0.15)	0.18±0.03 (0.12;0.22)
TEN	0.30±0.04 (0.21;0.38)	0.45±0.03 (0.40;0.05)	MW	0.30±0.08 (0.15;0.45)	W12	0.53±0.07 (0.39;0.65)	-
BRE	0.09±0.03 (0.02;0.14)	0.16±0.04 (0.08;0.16)	LoW	0.57±0.12 (0.33;0.81)	W18	0.32±0.07 (0.18;0.44)	-
VOC	0.35±0.08 (0.20;0.49)	0.43±0.06 (0.30;0.55)	MidW	0.30±0.08 (0.15;0.45)	SCW	0.24±0.10 (0.07;0.43)	0.13±0.04 (0.04;0.21)
REACT	0.21±0.04 (0.13;0.27)	0.41±0.02 (0.37;0.44)	NW	0.18±0.04 (0.10;0.25)	SC12	0.67±0.15 (0.36;0.93)	-
FT	0.35±0.06 (0.23;0.48)	0.55±0.02 (0.50;0.59)	WH	0.27±0.07 (0.14;0.39)	SC18	0.53±0.12 (0.29;0.76)	-
-	-	-	WHORL	0.20±0.06 (0.08;0.32)	REA12	0.48±0.12 (0.24;0.70)	-
-	-	-	-	-	REA18	0.36±0.09 (0.18;0.52)	-

Values are presented as mean±standard deviation.

*h*^2^*_d_*, direct heritability estimate; *t*, repeatability; *h*^2^*_m_*, maternal heritability estimate; MOV, movement; TEN, tension; BRE, breathing; VOC, vocalization; REACT, reactivity; FT, flight time; LW, facial whorl on the left side; MW, facial whorl in the middle; LoW, low whorl; MidW, mid whorl; NW, number of facial whorls; WH, whorl height; WHORL, absence or presence of facial whorls; WW, weaning weight; W12 and W18, body weight at 12 and 18 months of age; SCW, scrotal circumference at weaning; SC12 and SC18, scrotal circumference at 12 and 18 months of age; REA12 and REA18, ribeye area at 12 and 18 months of age.

**Table 2:** Estimates of genetic correlations and highest posterior density (in brackets) of productive, reproductive, and carcass traits with temperament traits in Canchim cattle

Traits	MOV	TEN	BRE	VOC	REACT	FT
WW	0.02±0.11 (−0.23;0.20)	0.08±0.12 (−0.14;0.32)	0.11±0.25 (−0.15;0.98)	−0.71±0.32^*^ (−1.00;−0.11)	−0.09±0.15 (−0.37;0.20)	−0.24±0.13^*^ (−0.48;−0.03)
W12	0.02±0.11 (−0.20;0.22)	0.06±0.10 (−013;0.24)	0.32±0.25 (−0.18;0.83)	−0.71±0.32^*^ (−1.00;−0.08)	−0.04±0.14 (−0.29;0.24)	−0.26±0.14^*^ (−0.53;−0.01)
W18	0.03±0.13 (−0.20;0.30)	0.03±0.11 (−0.18;0.26)	0.32±0.30 (−015;0.98)	0.07±0.57 (−0.99;0.99)	0.00±0.16 (−0.31;0.30)	−0.09±0.19 (−0.45;0.29)
SCW	−0.27±0.55 (−1.00;0.47)	−0.27±0.44 (−1.00;0.20)	−0.24±0.33 (−0.87;0.35)	−0.09±0.24 (−0.64;0.34)	0.08±0.18 (−0.27;0.41)	0.19±0.25 (−0.64.0.30)
SC12	−0.17±0.68 (−1.00;0.97)	0.00±0.37 (−1.00;0.30)	0.09±0.25 (−0.40;0.56)	−0.03±0.34 (−0.54;0.51)	0.22±0.17 (−0.14;0.52)	0.26±0.25 (−0.19;0.76)
SC18	−0.12±0.38 (−1.00;0.27)	−0.03±0.34 (−1.00;0.32)	−0.01±0.26 (−0.55;0.47)	−0.10±0.29 (−0.65;0.52)	−0.04±0.19 (−0.41;0.34)	0.36±0.27 (−0.18;0.84)
REA12	0.06±0.14 (−0.22;0.30)	0.04±0.11 (−0.19;0.23)	0.16±0.23 (−0.31;0.63)	−0.02±0.11 (−0.48;0.47)	−0.51±0.30^*^ (−0.99;−0.10)	−0.44±0.15^*^ (−0.73;−0.14)
REA18	0.12±0.13 (−0.13;0.36)	0.21±0.13 (−0.01;0.45)	0.29±0.22 (−0.16;0.70)	0.11±0.24 (−0.34;0.60)	0.19±0.15 (−0.07;0.50)	−0.08±0.18 (−0.41;0.27)

Values are presented as mean±standard deviation.

* Genetic correlations that presented estimates within the highest density intervals and that did not include zero values.

MOV, movement; TEN, tension; BRE, breathing; VOC, vocalization; REACT, reactivity; FT, flight time; WW, weaning weight; W12 and W18, body weight at 12 and 18 months of age; SCW, scrotal circumference at weaning; SC12 and SC18, scrotal circumference at 12 and 18 months of age; REA12 and REA18, ribeye area at 12 and 18 months of age.

**Table 3:** Estimates of phenotypic correlations and highest posterior density (in brackets) of productive, reproductive, and carcass traits with temperament traits in Canchim cattle

Traits	MOV	TEN	BRE	VOC	REACT	FT
WW	0.05±0.08 (−0.09;0.27)	0.04±0.09 (−0.14;0.21)	−0.21±0.20 (−0.59;0.18)	−0.88±0.03^*^ (−0.93;−0.81)	−0.08±0.08 (−0.23;0.04)	−0.15±0.09^*^ (−0.30;−0.05)
W12	0.06±0.07 (−0.08;0.19)	−0.01±0.09 (−0.18;0.17)	−0.17±0.17 (−0.54;0.16)	−0.88±0.03^*^ (−0.93;−0.81)	0.03±0.07 (−0.10;0.16)	−0.13±0.08^*^ (−0.29;−0.03)
W18	−0.07±0.08 (−0.22;0.08)	−0.08±0.11 (−0.29;0.14)	−0.21±0.20 (−0.59;0.18)	−0.91±0.03^*^ (−0.96;−0.84)	0.05±0.08 (−0.19;0.08)	−0.04±0.09 (−0.21;0.14)
SCW	−0.07±0.17 (−0.40;0.28)	0.00±0.12 (−0.25;0.21)	−0.20±0.21 (−0.58;0.21)	−0.05±0.21 (−0.44;0.39)	−0.02±0.10 (−0.22;0.16)	−0.05±0.08 (−0.19;0.11)
SC12	0.15±0.13 (−0.11;0.38)	0.04±0.17 (−0.25;0.35)	0.02±0.23 (−0.41;0.44)	0.11±0.31 (−0.54;0.64)	0.06±0.12 (−0.18;0.28)	0.19±0.15 (−0.10;0.46)
SC18	−0.14±0.14 (−0.40;0.15)	−0.08±0.18 (−0.44;0.24)	−0.20±0.24 (−0.66;0.29)	0.22±0.32 (−0.51;0.72)	−0.14±0.14 (−0.38;0.09)	0.03±0.14 (−0.24;0.26)
REA12	0.02±0.11 (−0.21;0.20)	−0.02±0.19 (−0.39;0.32)	−0.08±0.24 (−0.52;0.38)	−0.08±0.48 (−0.65;0.71)	−0.29±0.20 (−0.74;0.03)	−0.21±0.10^*^ (−0.42;−0.01)
REA18	0.06±0.10 (−0.12;0.25)	−0.26±0.14 (−0.53;0.38)	−0.06±0.42 (−0.60;0.78)	0.65±0.13 (−0.40;0.89)	0.07±0.10 (−0.11;0.26)	0.00±0.09 (−0.19;0.16)

Values are presented as mean±standard deviation.

* Genetic correlations that presented estimates within the highest density intervals and that did not include zero values.

MOV, movement; TEN, tension; BRE, breathing; VOC, vocalization; REACT, reactivity; FT, flight time; WW, weaning weight; W12 and W18, body weight at 12 and 18 months of age; SCW, scrotal circumference at weaning; SC12 and SC18, scrotal circumference at 12 and 18 months of age; REA12 and REA18, ribeye area at 12 and 18 months of age.

**Table 4:** Estimates of genetic correlations and highest posterior density (in brackets) of productive, reproductive, and carcass traits with facial hair whorl traits in Canchim cattle

Traits	LW	MW	LoW	MidW	NW	WH	WHORL
WW	0.20±0.19 (−0.14;0.60)	0.03±0.23 (−0.39;0.48)	0.20±0.19 (−0.14;0.60)	0.03±0.23 (−0.39;0.48)	0.07±0.21 (−0.32;0.48)	0.27±0.17 (−0.06;0.59)	0.20±0.20 (−0.19;0.59)
W12	−0.06±0.15 (−0.36;0.21)	−0.01±0.24 (−0.51;0.42)	−0.06±0.15 (−0.36;0.21)	−0.09±0.21 (−0.48;0.32)	−0.06±0.21 (−0.47;0.33)	0.10±0.18 (−0.25;0.43)	−0.08±0.19 (−0.44;0.29)
W18	−0.20±0.20 (−0.55;0.20)	−0.13±0.24 (−0.59;0.34)	−0.20±0.20 (−0.55;0.20)	−0.13±0.24 (−0.59;0.34)	−0.23±0.22 (−0.64;0.21)	0.25±0.20 (−0.11;0.66)	0.21±0.23 (−0.22;0.65)
SCW	0.38±0.26 (−0.15;0.83)	0.38±0.26 (−0.15;0.83)	0.38±0.26 (−0.15;0.83)	−0.24±0.29 (−0.73;0.37)	−0.06±0.31 (−0.62;0.55)	−0.63±0.42 (−0.98;0.44)	−0.06±0.42 (−1.00;0.62)
SC12	0.19±0.22 (−0.19;0.63)	−0.57±0.23^*^ (−1.00;−0.15)	0.19±0.22 (−0.19;0.63)	−0.57±0.23^*^ (−1.00;−0.15)	−0.41±0.23^*^ (−0.83;−0.02)	0.01±0.32 (−0.64;0.55)	0.13±0.44 (−0.61;1.00)
SC18	0.02±0.26 (−0.50;0.50)	−0.74±0.18^*^ (−0.99;−0.41)	0.02±0.26 (−0.50;0.50)	−0.74±0.19^*^ (−1.00;−0.40)	−0.74±0.18^*^ (−0.99;−0.40)	−0.14±0.27 (−0.65;0.39)	−0.29±0.30 (−0.82;0.30)
REA12	0.03±0.21 (−0.38;0.42)	−0.21±0.22 (−0.63;0.18)	0.03±0.21 (−0.38;0.42)	−0.21±0.22 (−0.63;0.18)	−0.15±0.21 (−0.52;0.27)	0.12±0.28 (−0.39;0.66)	−0.13±0.23 (−0.56;0.31)
REA18	0.00±0.20 (−0.40;0.38)	0.02±0.23 (−0.42;0.46)	0.00±0.20 (−0.40;0.38)	0.02±0.23 (−0.42;0.46)	0.05±0.21 (−0.38;0.44)	0.17±0.22 (−0.22;0.62)	−0.03±0.26 (−0.53;0.45)

Values are presented as mean±standard deviation.

* Genetic correlations that presented estimates within the highest density intervals and that did not include zero values.

LW, facial whorl on the left side; MW, facial whorl in the middle; LoW, low whorl; MidW, mid whorl; NW, number of facial whorls; WH, whorl height; WHORL, absence or presence of facial whorls, WW, weaning weight; W12 and W18, body weight at 12 and 18 months of age; SCW, scrotal circumference at weaning; SC12 and SC18, scrotal circumference at 12 and 18 months of age; REA12 and REA18, ribeye area at 12 and 18 months of age.

**Table 5:** Estimates of phenotypic correlations and highest posterior density (in brackets) of productive, reproductive, and carcass traits with facial hair whorl traits in Canchim cattle

Traits	LW	MW	LoW	MidW	NW	WH	WHORL
WW	0.04±0.03 (−0.01;0.10)	0.01±0.03 (−0.05;0.05)	0.04±0.03 (−0.01;0.10)	0.01±0.03 (−0.05;0.05)	0.01±0.03 (−0.04;0.06)	−0.03±0.47 (−0.72;0.78)	0.04±0.52 (−0.77;0.85)
W12	0.02±0.03 (−0.03;0.08)	−0.02±0.03 (−0.03;0.07)	0.02±0.03 (−0.03;0.08)	−0.01±0.03 (−0.04;0.07)	0.01±0.13 (−0.04;0.07)	0.02±0.04 (−0.70;0.69)	0.00±0.45 (−0.69;0.82)
W18	−0.02±0.03 (−0.08;0.04)	−0.03±0.03 (−0.09;0.02)	−0.02±0.03 (−0.08;0.04)	−0.03±0.03 (−0.09;0.02)	−0.04±0.03 (−0.10;0.02)	0.14±0.46 (−0.66;0.84)	0.10±0.53 (−0.74;0.86)
SCW	0.07±0.04 (−0.02;0.15)	0.07±0.04 (−0.02;0.15)	0.07±0.04 (−0.02;0.15)	0.05±0.04 (−0.03;0.12)	0.04±0.04 (−0.03;0.12)	−0.44±0.39 (−0.96;0.33)	0.00±0.44 (−0.72;0.73)
SC12	0.06±0.05 (−0.04;0.15)	−0.03±0.04 (−0.11;0.05)	0.0.6±0.05 (−0.04;0.15)	−0.03±0.04 (−0.11;0.05)	−0.03±0.04 (−0.11;0.05)	0.01±0.35 (−0.65;0.63)	0.09±0.41 (0.63;0.81)
SC18	0.04±0.05 (−0.05;0.13)	0.01±0.04 (−0.07;0.09)	0.04±0.05 (−0.05;0.13)	−0.12±0.35 (−0.83;0.43)	0.01±0.04 (−0.07;0.10)	0.03±0.36 (−0.69;0.62)	0.00±0.37 (−0.68;0.61)
REA12	0.03±0.04 (−0.04;0.10)	0.06±0.04 (−0.01;0.13)	0.03±0.04 (−0.04;0.10)	0.06±0.04 (−0.01;0.13)	0.05±0.04 (−0.02;0.11)	0.08±0.38 (−0.57;0.77)	−0.05±0.33 (−0.66;0.54)
REA18	0.06±0.04 (−0.01;0.13)	0.03±0.04 (−0.03;0.10)	0.06±0.04 (−0.01;0.13)	0.03±0.04 (−0.03;0.10)	0.04±0.04 (−0.02;0.11)	0.13±0.37 (−0.53;0.75)	0.08±0.46 (−0.73;0.74)

Values are presented as mean±standard deviation.

LW, facial whorl on the left side; MW, facial whorl in the middle; LoW, low whorl; MidW, mid whorl; NW, number of facial whorls; WH, whorl height; WHORL, absence or presence of facial whorls; WW, weaning weight; W12 and W18, body weight at 12 and 18 months of age; SCW, scrotal circumference at weaning; SC12 and SC18, scrotal circumference at 12 and 18 months of age; REA12 and REA18, ribeye area at 12 and 18 months of age.

## Data Availability

Upon reasonable request, the datasets of this study can be available from the corresponding author.

## SUPPLEMENTARY MATERIAL

**Supplement 1**. Fixed effects included in the genetic evaluation model.

**Supplement 2**. Descriptive statistics of temperament and facial hair whorl traits.

**Supplement 3**. Descriptive statistics of productive, reproductive and carcass traits.

**Supplement 4**. Estimates of genetic correlations (± standard deviation) between productive, reproductive, and carcass traits.
