# Supplementary material for: Genetic associations of temperament and facial hair whorls with productive, reproductive, and carcass traits in Canchim beef cattle
Source: Anim Biosci. 2026 Apr 28;39(10):260151. doi: 10.5713/ab.260151 (PMC13662933; doi:10.5713/ab.260151)
Supplement: Supplementary file 1 [file ab-260151-Supplementary-1.pdf]

SUPPLEMENTARY MATERIAL

Supplement 1. Fixed effects included in the genetic evaluation model

Traits		Fixed effect							CG
		Sex	Year of birth	Month of birth	Lineage[*]	EO[§]	Period[¥]	Age[#]	
Temperament	MOV	X	X	X	-	X	-	X	63
	TEN	X	X	X	-	-	-	X	63
	BRE	X	X	X	-	-	-	X	56
	VOC	-	X	-	X	-	-	-	50
	REACT	X	X	X	-	-	-	X	74
	FT	X	X	X	-	-	-	X	44
Facial hair whorls	LW	X	X	X	-	-	-	-	43
	MW	X	X	X	-	-	-	-	43
	RW	X	X	X	-	-	-	-	43
	LoW	X	X	X	-	-	-	-	43
	MidW	X	X	X	-	-	-	-	43
	HiW	X	X	X	-	-	-	-	43
	NW	X	X	X	-	-	-	-	43
	WH	X	X	X	-	-	-	-	43
	WHORL	X	X	X	-	-	-	-	43
Productive	WW	X	X	-	-	-	-	X	20
	W12	X	X	-	-	-	-	X	20
	W18	X	X	-	X	-	-	X	27
Reproductive	SCW	-	X	-	-	-	-	X	10
	SC12	-	X	-	-	-	-	X	7
	SC18	-	X	X	-	-	-	X	13
Carcass	REA12	X	X	-	-	-	-	X	8
	REA18	X	X	-	X	-	-	X	14

MOV = movement, TEN = tension, BRE = breathing, VOC = vocalization, REACT = reactivity, FT = flight time, LW = facial whorl on the left side, MW = facial whorl in the middle, RW = facial whorl on the right side, LoW = low whorl, MidW = mid whorl, HiW = high whorl, NW = number of facial whorls, WH = whorl height, WHORL = absence or presence of facial whorls, WW = weaning weight, W12 and W18 = body weight at 12 and 18 months of age, SCW = scrotal circumference at weaning, SC12 and SC18 = scrotal circumference at 12 and 18 months of age, REA12 and REA18 = ribeye area at 12 and 18 months of age, CG = contemporary group, X = fixed effect included, - = fixed effect not included, *Lineage = old, new and crossbred, § EO = entry order of the batches for temperament assessment, ¥ Period = assessments performed in the morning and afternoon, # Age = age at measurement as covariate.
