# Supplementary material for: Genetic associations of temperament and facial hair whorls with productive, reproductive, and carcass traits in Canchim beef cattle
Source: Anim Biosci. 2026 Apr 28;39(10):260151. doi: 10.5713/ab.260151 (PMC13662933; doi:10.5713/ab.260151)
Supplement: Supplementary file 2 [file ab-260151-Supplementary-2.pdf]

Supplement 2. Descriptive statistics of temperament and facial hair whorl traits

Temperament

Traits	Number		Score in %					Mean	Mode	Median
	Animals	Measures	1	2	3	4	5			
MOV	1,791	3,880	40	34	13	9	4	2.01	1	3
TEN	1,794	3,934	42	38	19	1	-	1.77	1	2
BRE	1,633	3,771	78	22	-	-	-	1.22	1	1
VOC	1,419	3,438	97	3	-	-	-	1.04	1	1
REACT	2,061	4,449	-	-	-	-	-	55.47	45.25	48.14
FT*	1,231	2,941	-	-	-	-	-	1.95	0.92	1.91

Facial hair whorls

Traits	Number of animals	Frequency (%)					Mean	Mode	Median
		1	2	3	4	5			
LW	1,505	11	89	-	-	-	1.88	2	2
MW	1,506	84	16	-	-	-	1.16	1	1
RW	1,506	9	91	-	-	-	1.88	2	2
LoW	1,505	11	89	-	-	-	1.88	2	2
MidW	1,506	84	16	-	-	-	1.16	1	1
HiW	1,505	10	90	-	-	-	1.88	2	2
NW	1,511	28	60	10	2	-	1.15	2	2
WH	1,286	14	8	41	18	19	3.24	3	3
WHORL	1,437	12	88	-	-	-	1.88	2	2

MOV = movement, TEN = tension, BRE = breathing, VOC = vocalization, REACT = reactivity, FT = flight time, LW = facial whorl on the left side, MW = facial whorl in the middle, RW = facial whorl on the right side, LoW = low whorl, MidW = mid whorl, HiW = high whorl, NW = number of facial whorls, WHORL = absence or presence of facial whorls, WH = whorl height, *hundredths of a second.
