# Supplementary material for: Genetic associations of temperament and facial hair whorls with productive, reproductive, and carcass traits in Canchim beef cattle
Source: Anim Biosci. 2026 Apr 28;39(10):260151. doi: 10.5713/ab.260151 (PMC13662933; doi:10.5713/ab.260151)
Supplement: Supplementary file 3 [file ab-260151-Supplementary-3.pdf]

Supplement 3. Descriptive statistics of productive, reproductive and carcass traits

Traits	Number of animals	Mean	Standard deviation	Minimum	Maximum
WW (kg)	2,242	212.53	32.71	125.00	298.00
W12 (kg)	1,705	221.17	30.59	160.00	320.00
W18 (kg)	1,575	296.43	43.10	201.00	425.00
SCW (cm)	1,144	19.03	1.94	15.00	24.00
SC12 (cm)	545	22.29	2.99	17.00	30.00
SC18 (cm)	743	28.25	3.30	20.00	36.00
REA12 (cm^2)	771	34.64	6.58	21.10	51.60
REA18 (cm^2)	947	43.40	7.72	25.50	64.90

WW = weaning weight, W12 and W18 = body weight at 12 and 18 months of age, SCW = scrotal circumference at weaning, SC12 and SC18 = scrotal circumference at 12 and 18 months of age, REA12 and REA18 = ribeye area at 12 and 18 months of age.
