# Supplementary material for: Genetic associations of temperament and facial hair whorls with productive, reproductive, and carcass traits in Canchim beef cattle
Source: Anim Biosci. 2026 Apr 28;39(10):260151. doi: 10.5713/ab.260151 (PMC13662933; doi:10.5713/ab.260151)
Supplement: Supplementary file 4 [file ab-260151-Supplementary-4.pdf]

Supplement 4. Estimates of genetic correlations (± standard deviation) between productive, reproductive, and carcass traits

Traits	W12	W18	SCW	SC12	SC18	REA12	REA18
WW	0.90±0.09	0.85±0.09	0.56±0.11	0.29±0.15	0.24±0.18	0.73±0.09	0.04±0.17
W12	-	0.93±0.03	0.65±0.15	0.45±0.12	0.45±0.15	0.52±0.11	0.31±0.14
W18	-	-	0.38±0.20	0.49±0.18	0.51±0.16	0.84±0.21	0.43±0.16
SCW	-	-	-	0.91±0.07	0.81±0.09	0.29±0.26	-0.46±0.34
SC12	-	-	-	-	0.92±0.07	0.71±0.18	-0.10±0.32
SC18	-	-	-	-	-	0.72±0.17	0.17±0.27
REA12	-	-	-	-	-	-	0.90±0.09

WW = weaning weight, W12 and W18 = body weight at 12 and 18 months of age, SCW = scrotal circumference at weaning, SC12 and SC18 = scrotal circumference at 12 and 18 months of age, REA12 and REA18 = ribeye area at 12 and 18 months of age.
